# Lipid Metabolism Alteration by Endocrine Disruptors in Animal Models: An Overview

**DOI:** 10.3389/fendo.2018.00654

**Published:** 2018-11-08

**Authors:** Francesca Maradonna, Oliana Carnevali

**Affiliations:** ^1^Dipartimento Scienze della Vita e dell'Ambiente, Università Politecnica delle Marche, Ancona, Italy; ^2^INBB Consorzio Interuniversitario di Biosistemi e Biostrutture, Rome, Italy

**Keywords:** phthalates, zebrafish (*Danio rerio*), metabolic disorders, epigenetic, reproduction

## Abstract

Exposure to potential Endocrine Disrupting Chemicals (EDCs) pose a documented risk to both wildlife and human health. Many studies so far described declining sperm counts, genital malformations, early puberty onset, highlighting the negative impact on reproduction caused by the exposure to many anthropogenic chemicals. In the last years, increasing evidence suggested that these compounds, other than altering reproduction, affect metabolism and induce the onset of obesity and metabolic disorders. According to the “environmental obesogens” hypothesis, evidence exists that exposure to potential EDCs during critical periods when adipocytes are differentiating, and organs are developing, can induce diseases that manifest later in the life. This review summarizes the effects occurring at the hepatic level in different animal models, describing morphological alterations and changes of molecular pathways elicited by the toxicant exposure. Results currently available demonstrated that these chemicals impair normal metabolic processes via interaction with members of the nuclear receptor superfamily, including steroid hormone receptors, thyroid hormone receptors, retinoid X receptors, peroxisome proliferator–activated receptors, liver X receptors, and farnesoid X receptors. In addition, novel results revealed that EDC exposure can either affect circadian rhythms as well as up-regulate the expression of signals belonging to the endocannabinoid system, in both cases leading to a remarkable increase of lipid accumulation. These results warrant further research and increase the interest toward the identification of new mechanisms for EDC metabolic alterations. The last part of this review article condenses recent evidences on the ability of potential EDCs to cause “transgenerational effects” by a single prenatal or early life exposure. On this regard, there is compelling evidence that epigenetic modifications link developmental environmental insults to adult disease susceptibility. This review will contribute to summarize the mechanisms underlying the insurgence of EDC-induced metabolic alterations as well as to build integrated strategies for their better management. In fact, despite the large number of results obtained so far, there is still a great demand for the development of frameworks that can integrate mechanistic and toxicological/epidemiological observations. This would increase legal and governmental institution awareness on this critical environmental issue responsible for negative consequences in both wild species and human health.

## Introduction

The first definition of Endocrine disrupting chemicals (EDCs) was provided at the European Workshop on endocrine disruptors (EDs) hold in Weybridge UK, in 1996. “An endocrine disruptor is an exogenous substance that causes adverse health effects in an intact organism, or its progeny, secondary to changes in endocrine function.” A further definition was also agreed, concerning potential EDs: “A potential endocrine disruptor is a substance that possesses properties that might be expected to lead to endocrine disruption in an intact organism.”

EDCs, interfering with the endocrine (or hormonal) system, are tightly implicated in the global decline of metabolic health. This large and multifaceted family includes plasticizers as phthalates, bisphenols, industrial chemicals including alkylphenols, flame retardants, air pollutants, such as polycyclic aromatic hydrocarbons, pesticides, metals, and dioxins. Consumption of contaminated food and water and inhalation of airborne pollutants represent the major sources of human exposure to EDCs, significantly contributing to the onset of obesity (1) by inappropriately stimulating adipogenesis as well as perturbing lipid metabolism and energy balance (2). All those EDCs that inappropriately regulate and promote lipid accumulation and adipogenesis are defined “obesogens” (3). In the last years, with the addition of compounds able to affect lipid metabolism, the list of obesogenic compounds significantly enlarged. Exposure to EDCs, in fact, can directly increase the size/number of adipocytes or indirectly affect basal metabolic rate and hormonal control of appetite (4). The hypothalamic-pituitary-adrenal axis plays an important role in controlling appetite and satiety, stimuli regulated by a variety of monoaminoergic, peptidergic and endocannabinoid (EC) signals that can be generated in the digestive tract, adipose tissue and brain. All these signals are candidate targets of potential obesogenic EDCs. Lipids, which role has been considered crucial in many tissues including liver, fat and intestine are accumulated and stored till their use in case of energy needs (5). Many studies so far have been carried out to reveal the pivotal role of dietary lipid intake as a source of essential fatty acids governing energy balance, food intake, growth, reproduction and health. Dysregulation of lipid accumulation is at the basis of several metabolic syndromes, including Nonalcoholic Fatty Liver Disease (NAFLD) and hyperlipidemia (6). The windows of exposure to potential EDCs (e.g., fetal or early postnatal) is critical for the outcome of metabolic diseases and results particularly detrimental because of the permanent effects on obesity later in life.

Since the socio-economic burden of EDC-caused diseases in industrialized countries ranges between 50 and 300 billion €/year (7), research to increase the knowledge on the causal link between health effects and EDCs represents a great challenge for health care systems.

This review is aimed at providing a general overview on the endocrine mechanisms linking EDC exposure to lipid metabolism dysregulation in different experimental vertebrate models, from mammals to fish, also considering *in vitro* trials.

## Potential mechanisms by which EDCs exert their effects

To better understand how potential EDCs can dysregulate lipid metabolism leading to the onset of several health diseases, a brief overview of the mechanisms and of the main actors involved in the regulation of lipid synthesis and degradation will be given.

In the last decades, the study of a group of xenobiotic compounds known as peroxisome proliferators has led to the discovery of peroxisome proliferator-activated receptors (PPARs) as a novel subfamily of nuclear receptors (NRs) (8). They dimerize with retinoid X receptor (RXR) and bind to PPAR-responsive DNA regulatory elements controlling the expression of genes involved in adipogenesis, glucose, lipid, and cholesterol metabolism (9, 10). Similarly to the membrane Estrogen receptorα (ERα), recently it has been demonstrated that PPARs can activate a non-genomic, rapid signaling pathway (11, 12), but while several studies so far described the activation of the ER non-genomic, rapid pathway in response to potential EDCs (13–16), information regarding ability of pollutants to activate the PPAR-non genomic signaling is still lacking.

**Figure 1:**
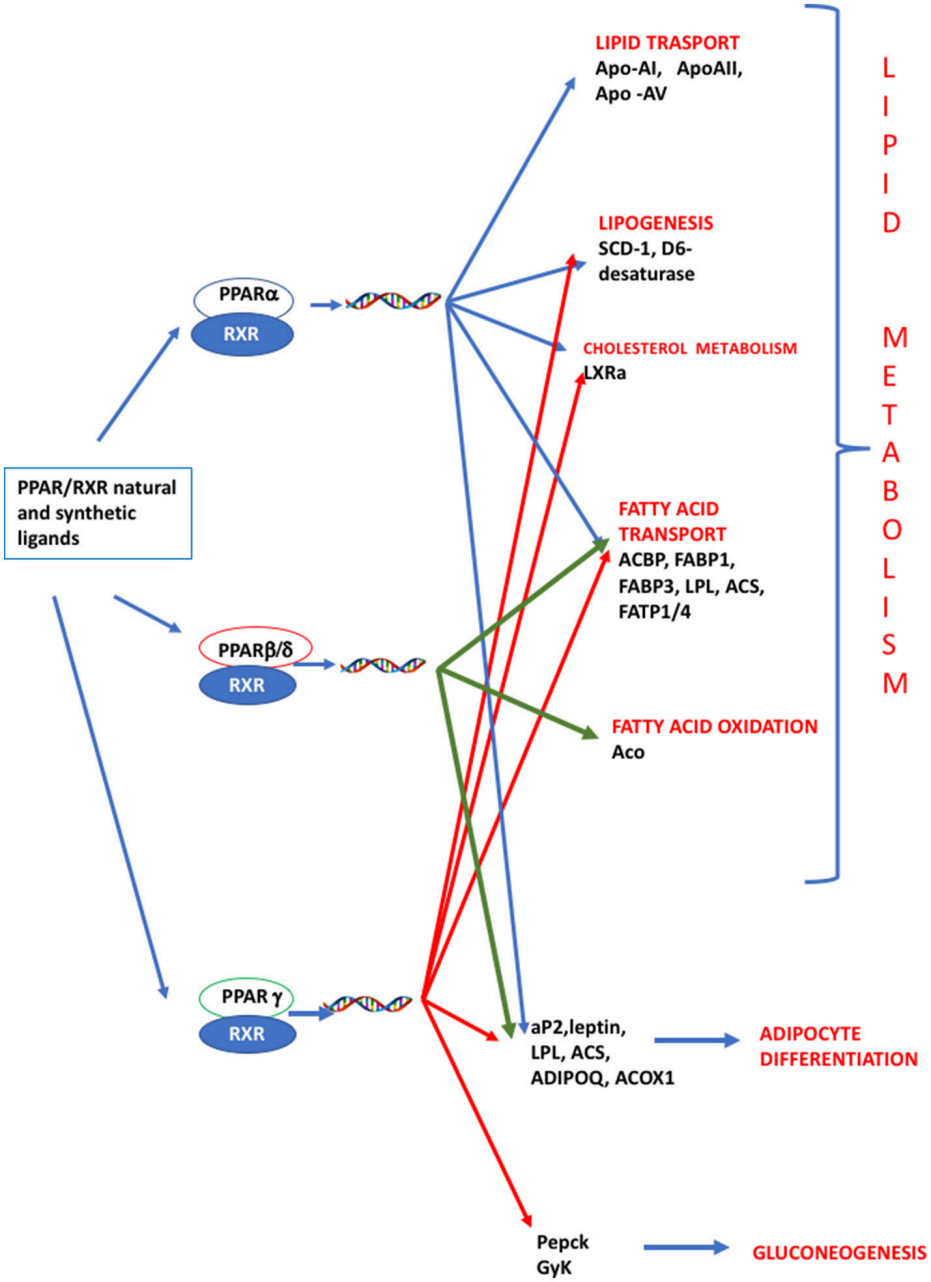
PPAR signaling pathway. PPARs are nuclear hormone receptors that are activated by fatty acids and their derivatives. PPAR α, δ/β, ɤ, show different expression patterns in vertebrates. Each of them is encoded by a separate gene and binds fatty acids, eicosanoids and synthetic ligands. Key genes are reported. PPARα/RXR heterodimer activates the transcription of genes involved in lipid metabolism, including transport, lipogenesis, cholesterol metabolism and adipocyte differentiation. PPARβ/RXR heterodimers activate the transcription of signal involved in fatty acid transport, fatty acid oxidation, and signal triggering final adipocyte differentiation. PPARɤ/RXR heterodimers are involved in different steps of lipid metabolism and regulate the transcription of signal responsible for adipocyte differentiation and gluconeogenesis. ACBP, Acyl-CoA-binding protein; ACS, Acetyl-coenzyme A synthetase; ACO, andacyl-CoA oxidase; ACOX1, Peroxisomal acyl-coenzyme A oxidase 1; ADIPOQ, adiponectin; aP2, adipocyte fatty acid binding protein 2; Apo-AI, apolipoprotein A1; ApoAII, apolipoprotein AII; Apo-AV, apolipoprotein AV; FABP1, fatty acid binding protein 1; FABP3, fatty acid binding protein 1; FATP1/4, Fatty acid transport protein 1–4; GyK, glycerol kinase; LPL, lipoprotein lipase; LXRα, Liver receptor α; Pepck, phosphoenolpyruvate carboxykinase; SCD-1 stearoyl-CoA desaturase-1.

**Figure 2:**
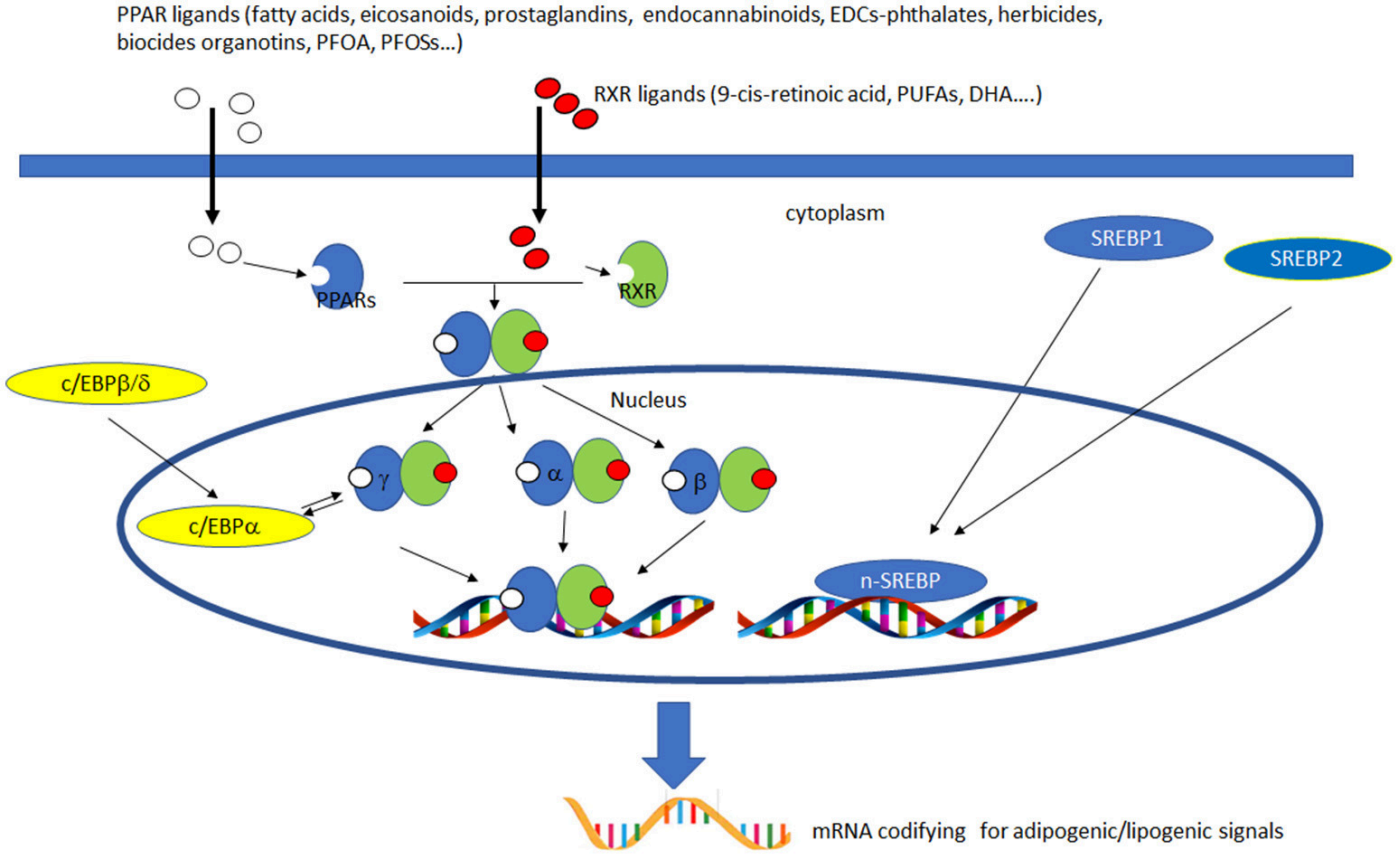
Activation of lipogenic and adipogenic pathways. PPARs (α, β/δ, and γ) belong to the nuclear hormone receptor superfamily and are ligand-activated transcription factors activated by fatty acids, fatty acid derivatives (e.g., eicosanoids), endocannabinoids and potential EDCs. PPAR and RXR dimers form important transcription activators which upon binding PPAR response elements can modulate many important cell functions, e.g., PPARα-RXR dimers activate genes controlling peroxisome proliferation, fatty acid metabolism and lipid homeostasis; PPARγ-RXR dimers affect adipocyte differentiation. C/EBPs are a family of nuclear activators, transiently expressed very early during adipocyte differentiation. C/EBPβ/δ activate the expression of of C/EBPa. Furthermore, the expression of C/EBPa and PPARγ is sustained by apositive feedback loop. Both proteins cooperatively promote downstream adipocyte-related genes transcription. SREBPs are activators of the complete program of hepatic cholesterol and fatty acid synthesis. SREBP-1 preferentially activates genes of fatty acid and triglyceride metabolism, whereas SREBP-2 preferentially activates genes of cholesterol metabolism. SCAP transports SREBPs from the ER to the Golgi apparatus, where is cleaved by two proteases, Site-1 protease (S1P) and Site-2 protease (S2P). nuclear SREBP (nSREBP), translocates to the nucleus, where it activates transcription of multiple target genes. SREBP-2 responsive genes include those for the enzymes HMG-CoA synthase, HMG-CoA reductase, farnesyl diphosphate synthase, and squalene synthase. SREBP-1 responsive genes include those for ATP citrate lyase and acetyl-CoA carboxylase and fatty acid synthase, the fatty acid elongase complex, (27) stearoyl-CoA desaturase, and glycerol-3-phosphate acyltransferase (28) Finally both SREBP forms activate three genes required to generate NADPH, which is consumed at multiple stages in these lipid biosynthetic pathways (29).

PPARs have a pivotal role in regulating metabolism, resulting the primary lipid sensors in vertebrates and being highly conserved between humans and zebrafish (17). Poly and mono unsaturated fatty acids (FA), eicosanoids and lipophilic hormones are PPAR natural ligands (18) with different affinity to PPAR isoforms and induce the expression of genes and enzymes involved in lipid metabolism. In addition to natural ligands, phthalates, plasticizers, certain herbicides, biocides organotins, perfluorooctanoic (PFOA) and perfluorooctanesulfonic (PFOS) acids, pharmaceuticals, halogenated derivatives of bisphenol A (BPA), the imidazole fungicide triflumizole, the fibrate class of hypolipidemic drugs, all listed as “Endocrine Disruptive Chemicals,” interact with the above stated NR through a specific binding mechanism (19). The activation of RXR- PPARα dimer stimulates FA β-oxidation (20), while the RXR-PPARɤ heterodimer favors preadipocytes differentiation and regulates lipid biosynthesis and storage (21) (Figure 1). In most species, the major site of both lipolytic and lipogenic processes is the liver, where PPARα and β, other than regulating FA β-oxidation, have a key role in glucose storage, lipoprotein capture and inflammation reduction (22), while the activation of PPARɤ orchestrates adipocyte function and differentiation as well as lipid storage within adipocytes (9). In the liver, PPARα is abundantly expressed, whereas PPARβ and PPARɤ are expressed at lower levels. PPARα, being the major regulator of the hepatic response to fasting, induces the expression of a variety of genes involved in FA catabolism and ketogenesis (23). Consequently, fasting PPARβ knockout mice develop hepatic steatosis (23). In addition to PPARɤ, several genes regulate fat cell development and control, including CCAAT-enhancer-binding proteins (*c/ebp*), responsible for the secretion of adipokines, e.g., leptin and adiponectin, hepatic glucose metabolism, insulin sensitivity and inflammation (24). A side from PPARs and C/EBP, sterol regulatory element-binding proteins (SREBPs) are central players in lipid metabolism, controlling the expression of genes important for lipid synthesis and uptake (25). In addition to the canonical functions in the transcriptional regulation of genes involved in lipid biosynthesis and uptake, SREBPs are also implicated in pathogenic processes including, inflammation, autophagy and apoptosis, and in this way, they contribute to the onset of several metabolic disorders (26). Activation of selected pathways responsible for adipogenesis and lipogenesis are summarized in Figure 2.

Once synthesized, lipids are stored within hepatocytes as source of energy. At the same time in these cells xenobiotic detoxification takes place. P450 enzymes are mainly implicated in this process via ligand-activated xenobiotic receptors, mainly aryl hydrocarbon receptor (AHR), constitutive androstane receptors (CAR) and pregnane X receptor (PXR). Recently, it was proposed that activation of these xenobiotic receptors is a triggering event of hepatic steatosis (30). To increase the knowledge regarding this aspect, it should be briefly considered the role of some signals involved in lipid regulation. In most species, triacylglycerol (TAG) is the main dietary component and lipoprotein lipase (LPL) is deputed to its hydrolyzation into non-esterified FA and 2-monoacylglycerol and their further storage in lipid droplets. It acts as “gate keeper” of FA uptake, working as a rate limiting enzyme in the provision of fatty acids to tissues (31). Intracellular transport of FA is performed by fatty acid binding proteins (FABP), which sequesters lipophilic compounds, regulates hepatocyte growth and transport them into mitochondria (32). Once stored in mitochondria, a set of genes involved in different aspects of lipid metabolism are activated (33). Fatty acid synthase (FAS) catalyzes the main pathway of lipogenesis, producing long saturated chain of carbon's atoms, finally stored in adipose tissues (34). Considering this brief state of art, it's clear that pivotal in this context is the ability of potential EDCs to interfere with/activate the PPAR cascade.

As novelty, recently, evidence emerged showing that one potential mechanism by which chemical exposure can influence lipid metabolism is through disturbance of circadian rhythms. While the circadian signals generated by clock genes produce metabolic rhythms, clock gene function is tightly coupled with fundamental metabolic processes, such as glucose and lipid metabolism (35). It has been demonstrated that the expression of clock genes, *Dec1, Dec2*, and *Bmal1*, is directly linked to energy metabolism, since *ppar* regulation is under the control of clock proteins. More specifically, DEC1 and DEC2 regulate adipogenesis by repressing the transcription of *ppar*ɤ (36).

In the last years, a direct link between the EC system and its role in the regulation of energy balance and in the onset of obesity emerged. ECs, differently from the protein hormone-based circuits and from the classical brain neurotransmitters, are synthesized and act locally and their effects are mediated by binding to surface receptors (37). Increasing evidence suggested that ECs bind and activate PPARs, being ECs fatty acids derivatives (38). It is likely that ECs, whose chemical structure is derived from arachidonic acid, might act not only through the classical type 1 and type 2 cannabinoid receptors (CB1 and CB2), the GPR55 orphan receptor and vanilloid type-1 receptor (VR1), but also through PPARs. Therefore, the binding between ECs and PPARs might mediate many of the biological effects of cannabinoids, including modulation of feeding behavior and lipid metabolism. Recent studies demonstrated the ability of potential EDCs to regulate the EC system. Among phthalates, the di-ethyl-hexyl-phthalate (DEHP) exerts its obesogenic action up-regulating hepatic *ppar*α*, cb1*, and *srebp* levels and stimulating *de novo* FA synthesis and hepatic steatosis. This hepatic state may cause an inhibition of food intake stimulus up-regulating leptin, the typical sensor of the energy status, which, in the brain, may negatively control *cb1* and in turn reduce *srebp* gene expression (25).

## Effects of EDCs exposure on lipid metabolism in animal models

### Insights on *in vitro* and *in vivo* exposure in

- **Mammalian Models**
  Increasing concern arose from the evidence that exposure to potential EDCs during critical periods, when adipocytes are differentiating and organs are developing, can induce effects, often as metabolic diseases, that manifest later in life. The correct functioning of the endocrine system has a central role in the organisms' health and its deregulation is directly responsible for the onset of many metabolic disorders, including obesity, NAFLD, and hyperlipidemia. The NAFLD, consisting of the excess triglyceride accumulation within hepatocytes, or steatosis, is considered the hepatic manifestation of obesity and metabolic syndromes. In addition, over the past four decades, research demonstrated that most of these metabolic diseases correlate, at least in part, with exposures to environmental chemicals (39). Potential EDCs can disrupt the normal hormonals level by inhibiting or stimulating the production of hormones or changing the way in which the hormones are transported to target tissues (4).
  This part of the review collects the most recent results relative to the *in vivo* or *in vitro* exposure of mammalian models to environmental chemicals.
  One of the first discovered synthetic PPAR ligand was tributyltin (TBT). Its exposure drives the differentiation of murine 3T3-L1 adipocytes *in vitro* and activates the RXR-PPARɤ-mediated pro-adipogenesis in liver and adipose tissue (40–42). In addition, an *in vivo* study, demonstrated that prenatal exposure to TBT, results in precocious lipid accumulation in adipose tissues and onset of hepatic steatosis in newborn mice (43). Adipogenesis was also promoted in human and mouse mesenchymal stem cells (MSCs) after a 14-days exposure to dibutyltin (DBT), the major TBT metabolite. More specifically, human MSCs resulted more responsive to the treatment than mouse MSC, with C/EBPα and PPARγ2, important signals in adipose differentiation and regulating each other through a positive feedback loop, resulting significantly up-regulated. In these cells, FABP4, fat-specific protein-27 (FSP27), and LPL were also over-expressed. DBT-induced adipogenic differentiation was abolished by the PPARγ antagonist T0070907, indicating that DBT was acting primarily through PPARγ (44). The same authors observed an impairment of glucose tolerance, driven by a hypothalamic resistance to leptin rather than to a misfunctioning of Langerhans islet in mice perinatally exposed to DBT, suggesting that DBT, as already observed for many other potential EDCs, can contribute to the diabetes epidemic (45). In Sprague-Dawley rats fed on a sucrose-high fat diet, nonylphenol (NP) co-administration, increased both water and food intake, hepatic echogenicity and alteration of several plasmatic aminotransferases. Hepatic macro-vesicular steatosis was found to be associated with congestion and dilation of central vein, inflammatory cell infiltration and up-regulation of genes involved in lipogenesis, e.g., *srebp-1C, fas*, and *ucp2* were described (46), suggesting that NP exposure exacerbates alcoholic fatty liver diseases.
  Triclosan (TCS) exposure at non-cytotoxic concentrations can induce lipid accumulation by decreasing adipocyte protein 2 (*ap2*), *lpl*, and adiponectin (adipoq) gene expression, in human MSCs (47). Exposure of rats via oral gavage to DEHP (0.05, 5, 500 mg/kg) induces varying degrees of hepatic steatosis, associated with inflammation, lipid peroxidation, oedema of the liver cells and hepatic damage (48). Further *in vitro* studies, using HepG2 cells aimed at understanding the potential mechanisms involved in DEHP-induced toxicity. Results showed that DEHP promotes lipid accumulation in cells and alters the level of superoxide dismutase (SOD) and malondialdehyde (MDA) disrupting the balance of oxidative stress. Lipid accumulation in hepatocytes was promoted by the activation the SREBP-1c and PPARα-signaling pathway (49). The obesogenic effect of a chronic exposure to 2,3,7,8-tetrachlorodibenzo-p-dioxin (TCDD) [1 μg/kg body weight (bw)/week] of adult C57BL/6J mice from 10 to 42 weeks old, resulted obesogenic in adult mice (7% in males and 8% in females). A gender specific effect was observed in the fat mass distribution, in adipose tissue and in the hepatic triglyceride accumulation, with female resulting more susceptible to the exposure than males, providing evidence of the gender and multiorgan effects of dioxin (50). Other authors, using the same mice strain, demonstrated that the inhibition of the AHR prevents the diet–induced obesity and fatty liver (51), suggesting that TCDD toxic/obesogenic effects can be avoided by blocking AHR. In the same strain (C57BL/6J), the pubertal male, when orally administered with a cocktail of 10 mg/kg/body weight (bw) cypermethrin (CYP), 100 mg/kg/bw atrazine (ATZ) and 0.1 mg/kg/bw 17α-ethynyestradiol (EE2) for 4 weeks followed by a high-energy diet (HD) for 8 weeks, reported alteration of the hepatic levels of transcriptional factors including PPARα, PPARɤ, and SREBP1C and their target genes related to FA synthesis and oxidation, respect to control mice fed only a HD. The results showed that early-life-stage exposure to environmental EDCs affected the homeostasis of hepatic glucose and FA metabolism at adulthood (52). To simulate human environmental exposure to BPA, 3T3-L1 pre-adipocytes were cultured for 3 weeks with 1 nM BPA. The exposure enhanced pre-adipocyte proliferation and anticipated the expression of master genes involved in lipid/glucose metabolism. Induced adipocytes are hypertrophic, displayed impaired insulin signaling and reduced glucose utilization concomitant to an increase of pro-inflammatory cytokine expression, supporting the hypothesis that BPA exposure, during sensitive stages of adipose tissue development, may cause adipocyte metabolic dysfunction and inflammation, thus increasing the risk of onset of obesity-related diseases late in life (53).
- **Amphibians**
  Few data are available describing the effects of potential EDCs exposure on lipid metabolism in amphibians. In 2006, Grün and collaborators described the effects of TBT exposure in *Xenopus*, showing its ability to activate RXR/PPARɤ pathways and suggesting the evolutionary conservation of these signals among vertebrates. They further investigated the effects of the exposure to environmentally relevant low doses of TBT (1–10 nM), the RXR-specific ligands LG100268 and AGN195203 (10–100 nM), troglitazone (0.1–1 M), and E_2_ (1–10 nM) on the developmental process of *X. laevis* tadpoles from stage 48 to metamorphosis. After TBT or RXR/PPARγ ligand exposure, they observed a dose-dependent increase in ectopic adipocyte formation around the gonads of both sexes. E_2_ treatment did not induced evident effects on adipogenesis/lipogenesis (43).
- **Teleosts**
  To date, many studies documented the toxic response of fish exposed to environmental pollutants. Due to their physicochemical properties, most of toxic effects of organic compounds are dependent on their bioaccumulation in the lipids of aquatic organisms. Therefore, there is an increasing interest to investigate the gene expression as well as the presence and activity of proteins involved in FA metabolism.

A recent paper in zebrafish contributed to increase the knowledge on the effects of the exposure to environmental TBT concentrations on lipid metabolism. Exposure to 10 and 50 ng/L TBT from pre-hatch to 9 months of age, altered body weight, hepatosomatic index and hepatic triglyceride abundance in a gender and dose related manner, with male resulting more sensitive than female. Furthermore, in male, TBT significantly affected the transcription of key factors and enzymes involved in adipogenesis and lipogenesis (PPARγ, SREBP1, FASn, 11β-HSD2, C/EBPβ, and DGAT2). In female, hepatomegaly was observed, associated to a subtle, not significant adipogenic response in the transcription of genes (54). Differently to TBT, the exposure of zebrafish to triclosan (TCS), an antimicrobial agent, impaired mRNA expression levels of β-oxidation transcripts and lipid β-oxidation genes, including *ppar*α*, cpt1, lpbe, cyp4a10*, and *aco* (55). Similarly, in adult male zebrafish, DEHP exposure disrupted metabolic processes in the liver, as demonstrated by alteration of five biological pathways: “FOXA2 and FOXA3 transcription factor networks,” “Metabolic pathways,” “metabolism of amino acids and derivatives,” “metabolism of lipids and lipoproteins,” and “fatty acid, triacylglycerol, and ketone body metabolism” (56).

In the same model, non-toxic concentrations of four different potential EDCs, TBT, tetrabrominated bisphenol A (TBBPA), tris (1,3-dichloroisopropyl) phosphate (TDCIPP) and benzophenone 3 (BP-3), selected on the bases of their ability to affect PPAR signaling, were added to rearing water from 9 to 14 dpf. All tested pollutants induced obesity, as visualized and quantified by fluorescent lipid staining in the trunk area between the gall bladder and the proximal intestine. In addition, exposed larvae were fed a standard diet, resulted in an even higher lipid accumulation than larvae fed a hypercaloric diet (HCD), suggesting that early exposure to toxicant, could affect fish metabolism later in life. Moreover, the ability of the above selected potential EDCs to affect the circadian rhythm was also demonstrated. Using a transgenic Tg (4xEbox:Luc) zebrafish larvae (57), a 24-h TBT exposure showed its ability to reduce the amplitude of oscillations and a prolongation of the period between maximum and minimum activity respect to transgenic-control fish. The period was even further prolonged by the TDCIPP. A loss of the characteristic oscillations was also observed in larvae exposed to TBBPA or BP-3. These authors concluded that clock activity could be modulated by excess fatty acids by activating PPARγ signaling (58) which specifically down-regulates the clock gene period1 (59) with a repression of the Clock/Bmal complex formation.

The ability of TBT and triphenyltin (TPT) to promote adipocytes differentiation was demonstrated in trout (60). Primary cultured adipocyte treated with TBT and TPT induced lipid accumulation and slightly enhanced PPARγ and C/EBPα protein expression, suggesting that the use of a primary adipocyte cell culture from this species is a valuable *in vitro* tool to estimate the capacity of different compounds and their synergism to interfere with adipocyte differentiation and lipid accumulation.

Several studies so far demonstrated that ECs are present in adipose tissue and other peripheral tissues involved in energy metabolism (i.e., liver and muscle), thus representing an additional clue to the understanding of adipose tissue functioning and possible onset of metabolic syndromes including obesity. Recent studies demonstrate the ability of pollutants to modulate the ECS (61, 62). In particular, in zebrafish, DEHP exerts its obesogenic action by up-regulating hepatic *ppar*α*, cb1*, and *srebp* levels and by stimulating *de novo* FA synthesis and hepatic steatosis. This hepatic state may cause an inhibition of food intake stimulus by the up-regulation of leptin, the typical sensor of the energy status, which, in the brain, may negatively control *cb1* and in turn reduce *srebp* gene expression (63). Similar results were obtained after an acute exposure of adult zebrafish to BPA. The observed hepatosteatosis was associated with an increase in the liver of EC levels and variations of catabolic and anabolic enzyme levels. In the same study, acute and chronic exposure of HHL-5 cells to BPA, induced triglyceride accumulation in a CB1 dependent manner, suggesting that BPA induced hepatosteatosis in zebrafish and human hepatocytes is mediated by the up-regulation of EC system (64). Similarly, a 3 weeks chronic exposure of adults to three BPA concentrations (5, 10, and 20 μg/L), altered the expression of a number of genes involved in the EC control of metabolism in liver and brain, as well as that of endogenous ECs and EC-like mediators. These changes were associated with an increased presence of hepatic lipid vacuoles, without changes in food intake and appetite regulation (61). BPA exposure affected lipid metabolism in a non-monotonic dose-related fashion. The lowest dose of BPA increased the storage of TAGs and promoted FA synthesis, while the highest concentration promoted the *de novo* lipogenesis and cholesterologenesis (65). Moreover, a chronic BPA-exposure impacted the miRNome in adult zebrafish and established an epigenome more susceptible to cancer development. After a 3 weeks exposure to 100 nM BPA, in the liver, 6,188 mRNAs and 15 miRNAs were differently expressed (*q* ≤ 0.1), uncovering signatures associated with NAFLD, oxidative phosphorylation, mitochondrial dysfunction and cell cycle, suggesting BPA potentiality to cause adverse health outcomes including cancer (66), and supporting previous studies evidencing the miRNA pivotal role in lipid synthesis, oxidation and related diseases (67). The expression of four miRNAs, miR-125b, miR-205, miR-142a, and miR-203a were significantly modulated by TCS in different experimental models both *in vivo* and *in vitro* (55), and resulted implicated in the downstream regulation of genes responsible for FA synthesis and metabolism. Moreover, in zebrafish, TCS was directly involved in the upstream regulation of miR-125b (68).

The results above described revealed the obesogenic effects of several potential EDCs, many of which are plasticizers and in the last years, the need to replace them, e.g., BPA and DEHP, with safer compounds, arose. Alternative candidates of DEHP included diethylene glycol dibenzoate (DGB) and di-isononylphthalate (DiNP). In a pilot study, a chronic exposure to five DGB concentrations (0.01; 0.1; 1; 10; 100 μg/L) affected hepatic lipid metabolism leading to increased lipid production and mobilization in a non-monotonic dose-related fashion. The lowest DGB concentrations (0.01 μg/L and 0.1 μg/L), increased *de novo* lipogenesis, cholesterol esters, TAG production and the possible conversion of lipids into apolipoprotein particles. A small reduction in *apoAla*, concomitant with the increase of apoliprotein mRNA codifying for a protein involved in very low-density lipoprotein and chylomicron production was also measured. Exposure to the highest concentrations (10 and 100 μg/L), increased *cebpa* levels, involved in adipocyte differentiation. Moreover, FT-IR analysis revealed that DGB exposure lead to changes in the biochemical composition of liver where the length of the aliphatic chains and phospholipid content increased (65).

To date, one study described the role of DiNP in the onset of pathophysiologies in adult zebrafish. In addition to an impairment of oogenesis, which will be described later on, an up-regulation of orexigenic and hepatosteatotic signals together with a deregulation of the peripheral ECS and lipid metabolism was observed. At central level, a deregulation of ECS components (62), suggested that DEHP replacement with DiNP should be carefully evaluated.

Moving to marine teleosts, seabream resulted so far an excellent experimental model for ecotoxicological studies. In a feeding trial, juvenile seabream were fed a diet contaminated with BPA (5 mg/kg bw or 50 mg/kg bw BPA) or alkylphenolic contaminants, NP (5 mg/kg bw or 50 mg/kg bw NP) or *tert-octylphenol* (t-OP) (5 mg/kg bw or 50 mg/kg bw t-OP). The diet caused alteration of liver morphology, showing moderate-severe lipid accumulation, loss of the cord structure, ceroid accumulation and hydropic change in most of fed fish (69, 70). These findings prompted analysis of the expression of the major molecules involved in lipid metabolism: *ppars, fas, lpl*, and *hsl*. The modulation of the different signals strongly suggested that lipid accumulation within hepatocytes was associated to a decrease of lipid mobilization, thus causing hepatosteatosis as documented by histological analysis (70, 71).

**Figure 3:**
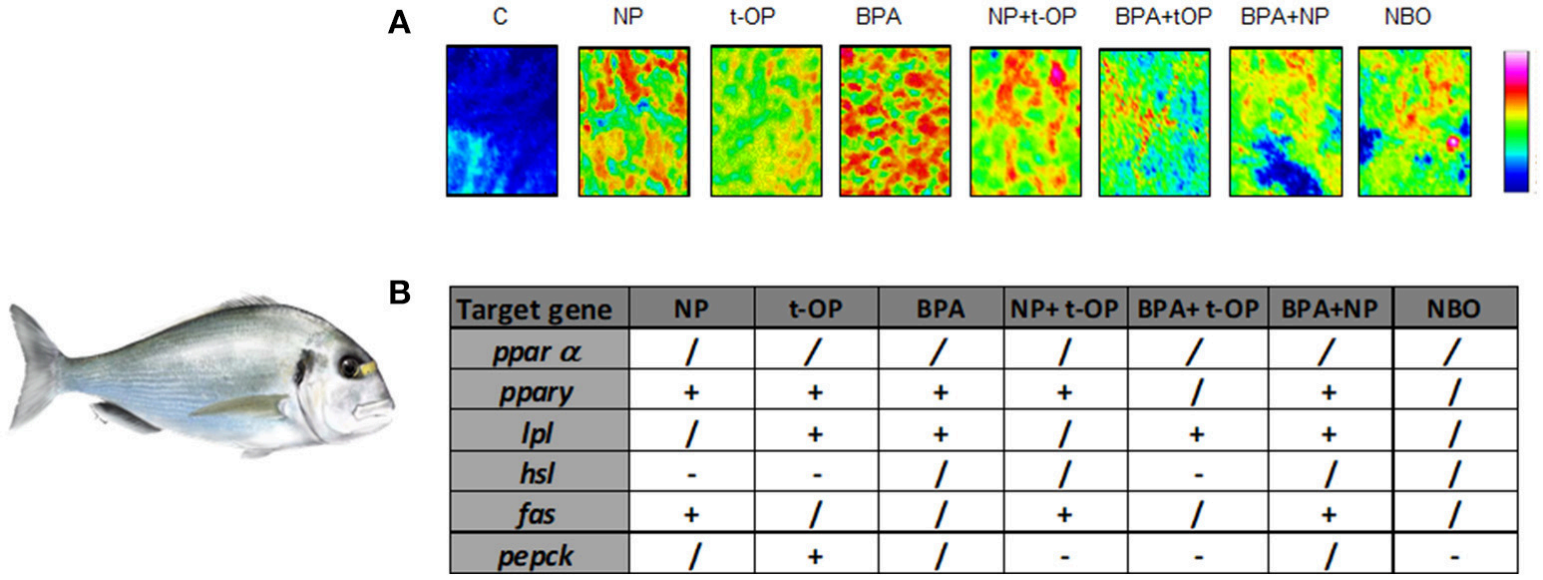
Modulation of lipid content and metabolism in Seabream fed xenobiotics. **(A)** False color images of liver sections from C, NP, t-OP, BPA, and xenobiotic mixtures representing the topographical distribution of lipids. Adapted from Carnevali et al. (72). **(B)** mRNA variations in the different experimental groups. “+” upregulation, “–” downregulation, “/” no changes respect to control values. Experimental groups C, control fish receiving the commercial feed; NP, fed the commercial feed enriched with 5 mg/kg bw NP; t-OP, fed the commercial feed enriched with 5 mg/kg bw t-OP; BPA, fed the commercial feed enriched with 5 mg/kg bw BPA; NP + t-OP, fed the commercial feed enriched with 5 mg/kg bw NP + 5 mg/kg bw t-OP; BPA + t-OP, fed the commercial feed enriched with 5 mg/kg bw BPA + 5 mg/kg bw t-OP; BPA + NP, fed the commercial feed enriched with 5 mg/kg bw BPA + 5 mg/kg bw NP; NBO, fed the commercial feed enriched with 5 mg/kg bw NP + 5 mg/kg bw BPA + 5 mg/kg bw t-OP. Seabream picture by Dr. Marco Graziano http://tiktaalikillustrations.com.

Since most of studies have focused on the effects induced by the exposure to a single compound, recently the attention moved to the combined effects of mixtures of substances with dissimilar modes of action. In a further study, seabream were fed on mixture of the above mentioned xenobiotics (NP, t-OP, BPA) and results evidenced that the administration of mixture of contaminants exerts a milder lipogenic effect, highlighting the contrasting/antagonistic interaction among chemicals (72) (Figure 3). In the same experimental model, a chronic exposure to two nominal concentrations of DGB (1 μg/L or 100 μg/L), demonstrated its action as PPARα agonist, resulting in a potential stimulation of key lipolytic genes and in a concomitant down-regulation of those involved in the ECS regulation (73). Using sea bream hepatocytes, the integration of *in silico* predictions with *in vitro* experiment results, evidenced the possible dose-relationship effects of diisodecyl phthalate (DiDP) exposure on PPAR:RXR-dependent gene expression pathways. Principal component analysis (PCA) showed the strength of relationship between transcription of most genes involved in FA metabolism and *ppar* mRNA levels. In particular, *fabp* was highly correlated to all *ppars* (74). Recently, in the same experimental model, it was demonstrated that dietary administration to BPA and DiNP altered the hepatic structure and the biochemical composition, increasing the presence of lipids and TAGs and decreasing phospholipids and glycogen abundance. In addition, the diet altered hepatic levels of ECs and EC-like mediators. These alterations were also associated to changes at the transcriptomic level of genes involved in lipid biosynthesis and ECS metabolism (75).

Marine medaka, *Oryzias javanicus*, male fish were exposed to 76 mg/L of BPA for 72 h to analyze the transcriptional responses of BPA exposure over four time-course by gene ontology enrichment analysis in terms of molecular functions. The most up-regulated transcripts belonged to lipid metabolism relevant genes, e.g., *apoA-IV, apoA-I*, long chain fatty acid CoA ligase (*acsl*), elongation of very long chain fatty acids protein (*elovl*), and *fabp*, showing that in medaka, BPA behaves in a similar way as in mammalian species (76).

Altogether, the results reported in this section, highlighted the common negative effects of the exposure to potential EDCs both in animal models and *in vitro* systems, providing a general overview on the toxicity of these environmental compounds, not depending from the route of exposure, via the food, via the water, perinatal, early in life or at adulthood.

## Transgenerational epigenetic inheritance: focus on the effects on adipogenesis and lipid metabolism signals

Recent studies revealed that the increasing incidence of obesity, in addition to being due to bad life styles and occupational stress, could be also caused by exposure to xenobiotics and transmitted to subsequent/following generations on an epigenetic inheritance base (77). To be called transgenerational, expression of the trait has to persist for at least two or three generations (three mammals, two fish) after the initial exposure to the environmental agent (78, 79). This section will describe the epigenetic mechanisms underlying developmental plasticity and uncovering the existence of a mechanistic link between altered epigenetic gene regulation following an early toxicant exposure and potential onset of obesity later in the life. In general terms, evidences demonstrated that the obesogenic effects of potential EDCs are mediated by their ability to bind NRs. These receptors directly recruit methyl and acetyltransferase, thus altering epigenetic marks regulating gene expression (80). In this way, EDCs can modify chromatin states or the levels of DNA or histone methyltransferases (81). As stated above, one of the main target of obesogens is PPARɤ, the master regulator of adipogenesis or its target-related genes. During the first developmental stages, PPARɤ regulates the differentiation of MSCs into osteocytes or adipocytes. An *in vitro* exposure of 3T3-L1 preadipocytes cell line to TBT, resulted in an increase of the number of differentiated adipocytes associated with a global decrease of DNA methylation (82). The same cell line exposed for 8-days to brominated diphenyl ether 47 (BDE-47), differentiated into adipocytes and presented higher levels of *ppar*ɤ*2, cebp*α*, cebp*β*, cebp*ɤ*, srebf1a, lpl, Slc2a4, fabp4, Adipoq, G6pc, Lep, and Igf1*. Variation of *ppar*ɤ*2* expression was associated with a decreased methylation of 3 CpG sites in promoter region (83). Moving to *in vivo* studies, in adipose-derived stem cells (ADSCs) isolated from white adipose tissue of C57BL/6J mice perinatally exposed to TBT by maternal gavage, increased lipid accumulation in differentiated adipocytes associated with an increase of early adipogenesis markers, *ppar*ɤ and *fapb4*, were measured. In these genes, hypomethylation of promoter/enhancer region of *fapb4* but not of *ppar*ɤ*2* were observed (82). Prenatally exposure of BALB/cByj mice (F1) to mixture of PAHs, displayed increased adult adipocyte size. The size alteration was also observed in F2-offspring and were associated in both F1 and F2, to general increased *ppar*ɤ*, cox2*, and *cebp*α expression in adipose tissue. In both males and females of F1 and F2, a decreased methylation of 1 CpG site in *ppar*ɤ promoter was detected, inversely correlating with *ppar*ɤ expression (84). In a transgenerational study, outbred gestating female rats were transiently exposed to DDT and the F1 generation offspring bred to generate the F2 generation and F2 generation bred to generate the F3 generation. The F1 and F3 generation were aged and various pathologies investigated. The transgenerational transmission of disease was through both female (egg) and male (sperm) germlines. F3 generation sperm epimutations and differential DNA methylation regions of a number of obesity-related genes were induced by DDT. Interestingly, in this study, the authors concluded that male obesity is transmitted through the female germline and female obesity transmitted through the male germline (78). In a study using rats, females were daily intraperitoneally injected with methoxychlor from days 8 to 14 of gestation and then the onset of disease was evaluated in adult F1 and F3 generation progeny. Increase of obese rate was observed especially in the F3 and F4 generation demonstrating that female germline transmission of environmentally induced epigenetic transgenerational phenotypes is equally as stable as male germline transmission (85). In another study, gestating female rats were transiently exposed from days 8 to 14 of embryo gonadal sex determination to a plasticizer mixture containing BPA, DEHP and dibutylphthalate (DBP) and the incidence of adult onset disease was evaluated in F1 and F3 generation rats. Obesity significantly increased in F3 rats (86). Epidemiological evidence showed that the developmental programming can be transferred to subsequent generation. The exposure to NP during critical windows of development, including fetal and/or early postnatal periods, can induce permanent alterations in adipose tissue and then obesity in mouse (87). NP action is mediated by ERα signaling pathway and the receptor deletion causes abdominal fat accumulation due to increased number and size of fat cells, increased levels of cholesterol and leptin and alteration in the expression of genes involved in lipogenesis and adipogenesis, in the two generation offspring's (88). Similarly to NP, it is well-known that BPA induces epigenetic modification (89, 90) and exerts its obesogenic action by binding ERα. The hormone-like-receptor complex bind the estrogen responsive element in the promoter of histone modifying methyltransferase EZH2 gene. After binding, several co-regulators are attracted and the up-regulation of the EZH2 levels increase H3K27 trimethylation (91). Recently Helsley and Zhou (92), reviewed the central role of PXR in lipid homeostasis. These evidences clearly suggest that although the research on obesogens mainly focuses on PPARɤ as master regulator, the involvement of other receptors, including steroid hormone receptor and PXR should be evaluated.

## EDC exposure: interplay between fat/lipid metabolism and reproduction

Body energy reserves are gated with reproduction and are sensitive to different metabolic signals. Main actors of this tight relationship between energy homeostasis and fertility are represented by metabolic hormones (ghrelin and leptin) and neuropeptides (kiss1 and kiss2) (93, 94), that regulate the levels and the release of Gonadotropin-releasing hormone (GnRH) (95). Thus, full activation of the hypothalamic-pituitary-gonadal axis at puberty and its proper functioning later at adulthood critically depends on adequate energy stores (96). The identification of the adipokine leptin, which transfers information on the body's metabolic status to hypothalamic centers governing reproduction, represents an important step to understand the mechanisms regulating this interplay (97). On this regard, of particular interest was the finding that DEHP, is able to modulate leptin expression in the liver of zebrafish showing the potentiality of this plasticizer to impair the entire endocrine system linking fat storage to appetite, energy expenditure and reproduction (63).

Using a multidisciplinary approach, ranging from qPCR analysis, to histology and Fourier transform infrared imaging (F-TIR), the effects of DiNP exposure were analyzed in zebrafish. Fish fecundity, oocyte growth, autophagic and apoptotic processes, as well as changes of morphological and biochemical composition of oocytes were investigated (98). Findings in zebrafish, provided evidence that exposure to DiNP adversely affects oocytes growth and maturation, leading to abnormal gonadal development and reproduction. More specifically, lipids, proteins and phosphate groups were significantly decreased in the ovaries of all the experimental groups and were associated to an alteration of vitellogenin, a phospholipoglycoprotein internalize by vitellogenic oocytes. These alteration of the macromolecular composition of oocytes and the decreased number of vitellogenic and mature eggs within the ovary could be responsible for the significant decrease of fecundity observed for all doses of DiNP (99). In Wistar rats receiving TBT for 15 days by gavage, metabolic dysfunctions and reproductive abnormalities were observed. The increase of leptin levels in obese rats, were negatively correlate with lower Kiss responsiveness, evidencing that TBT toxic effects may be either direct, on the reproductive axis, or indirect, by its abnormal metabolic regulation (100).

## Conclusion

In conclusion, this review summarizing the most recent results describing the effects of potential EDC administration on metabolic health, clearly evidences the risk caused by the exposure to environmental chemicals (Table 1). A summary of the most common lipid metabolism alterations following their exposure has been presented. In addition, novel data regarding their ability to affect circadian rhythms as well as to up-regulate the expression of the ECS, in both cases leading to a remarkable increase of lipid accumulation, have been also reported. Finally, evidences of their“transgenerational obesogenic effects” following a prenatal or early life contamination have been discussed.

**Table 1:** Main biological effects reported in different cell and animal models exposed to potential EDCs.

**Environmental pollutant**	**Animal model**	**Biological effect observed**	**Bibliography**
Benzophenone 3 (BP-3)	Zebrafish, *Danio rerio*	Obesity induction and alteration of circadian rhythms	(57)
Bisphenol A (BPA)	3T3-L1 pre-adipocytes	Adipocyte metabolic dysfunction and inflammation	(53)
	Zebrafish	Induction of TAG accumulation by up-regulation of ECS	(64)
	HHL-5 cells	Induction of TAG accumulation by up-regulation of ECS	(64)
	Zebrafish	Increased presence of hepatic lipid vacuoles, caused by alteration of the ECS	(61)
		Increased TAG storage and FA synthesis, *de novo* lipogenesis and cholesterologenesis promotion	(65)
		Evidence of the miRNome involvement in lipid synthesis, oxidation and related diseases	(67)
	Seabream, *Sparus aurata*	Hepatic lipid accumulation associated to a decrease of lipid mobilization	(70, 71)
		Alteration of hepatic structure lipids and TAG content and decreased phospholipids and glycogen abundance	(75)
	Marine medaka, *Oryzias javanicus*	Upregulation of *apo A-IV, apo A-I, Acsl1, Elovl, and fabp*	(76)
Brominated diphenyl ether 47 (BDE-47)	3T3-L1 pre-adipocytes	Increased levels of *Pparɤ2, Cebpα, Cebpβ, Cebpɤ, Srebf1a, Lpl, Slc2a4, Fabp4, Adipoq, G6pc, Lep, and Igf1*. *Transgenerational study*	(83)
Cypermethrin (CYP), atrazine (ATZ), 17α-ethynyestradiol (EE2)	C57BL/6J mice	Alteration of the hepatic levels of PPARα, PPARɤ, and SREBP1C	(52)
Dibutyltin (DBT)	Human MSCs	C/EBPα, PPARγ2, FABP4, FSP27, LPL upregulation	(44)
Dichlorodiphenyltrichloroethane (DDT)	Hsd:Sprague Dawley®™SD®™ Harlan	Obesity induction in males. *Transgenerational study*	(78)
Di(2-ethylhexyl) phthalate (DEHP)	HepG2 cells	Activation of the SREBP-1c and PPARα-signaling pathway	(49)
	Zebrafish	Alteration of FOXA2 and FOXA3 transcription factor networks', “Metabolic pathways,” “metabolism of amino acids and derivatives,” “metabolism of lipids and lipoproteins,” and “fatty acid, triacylglycerol, and ketone body metabolism”	(56)
		Up-regulation of hepatic PPARα, Cb1, and SREBP levels, *de novo* FA synthesis and hepatic steatosis	(63)
	Sprague-Dawley rats	Hepatic steatosis, associated to inflammation, lipid peroxidation, oedema of the liver cells and hepatic damage	(48)
Di-isodecyl- phthalate (DiDP)	Seabream	PPAR-mediated regulation of *fabp*	(74)
DiNP	Zebrafish	Upregulation of orexigenic and hepatosteatosis signals, deregulation of the peripheral and central ECS and lipid metabolism	(62)
DiNP	Seabream	Alteration of hepatic structure lipids and triglycerides content and decreased phospholipids and glycogen abundance	(75)
Diethylene glycol dibenzoate (DGB)	Zebrafish	Increase of de novo lipogenesis, cholesterol esters, TAG production and potential conversion of lipids into apolipoprotein particles	(65)
	Seabream	PPARα agonist, stimulation of key lipolytic genes and downregulation of ECS	(73)
Methoxychlor	Sprague-Dawley rats	Obesity induction in females. *Transgenerational study*	(85)
BPA, DEHP and dibutylphthalate (DBP) mixture	Sprague-Dawley rats	Obesity induction. Transgenerational study	(86)
Nonylphenol (NP)	Sprague-Dawley rats	Hepatic *srebp-1C, fas* and *ucp2 upregulation*	(46)
	Wistar rats	Obesity induction, increased levels of cholesterol and leptin and alteration of the expression of genes involved in lipogenesis and adipogenesis. *Transgenerational study*	(87, 88)
	Seabream	Hepatosteatosis, alteration of lipid metabolism	(69, 71)
BPA, NP, *tert-*octylphenol (*t*-OP) Mixture	Seabream	Alteration of lipid metabolism	(72)
Polycyclic aromatic hydrocarbon (PAH) mixture	BALB/cByj mice	Increased *Pparɤ, Cox2, and Cebpα* expression in adipose tissue. *Transgenerational study*	(84)
RXR-specific ligands LG100268 and AGN195203	African clawed frog, *Xenopus laevis*	Ectopic adipocyte formation around the gonads	(43)
Tributyltin (TBT)	murine 3T3-L1 adipocytes	RXR-PPARɤ-mediated pro-adipogenesis in liver and adipose tissue	(40, 41, 42)
		Increase the number of differentiated adipocites. *Transgenerational study*	(82)
	C57BL/6 mice	Lipid accumulation in adipose tissues and onset of hepatic steatosis	(43)
		Increased lipid accumulation in differentiated adipocytes associated to an increase of early adipogenesis markers, Pparɤ and Fapb4. *Transgenerational study*	(82)
	African clawed frog	Activation of RXR/PPARɤ pathways	(43)
	Zebrafish	Male: increased body weight, hepatosomatic index, hepatic TAG abundance and expression of adipogenesis and lipogenesis genes *pparγ, srebp1, fasn, 11β-hsd2, c/ebpβ*, and *dgat2* Female: hepatomegaly with lack of response of signals involved in adipogenesis	(54)
		Obesity induction and alteration of circadian rhythms	(57)
	Trout (*Oncorhynchus mykiss*) Primary adipocyte culture	Promotion of adipocytes differentiation by enhancing PPARγ and C/EBPα protein expression	(60)
2,3,7,8-Tetrachlorodibenzodioxin (TCDD)	C57BL/6J mice	Sex specific modulation of mRNA levels involved in adipose tissue and hepatic metabolism, inflammation, xenobiotic metabolism and endocrine disruption	(50)
		AHR mediates obesity and fatty liver onset	(51)
Tetrabrominated bisphenol A (TBBPA)	Zebrafish	Obesity induction and alteration of circadian rhythms	(57)
*t*-OP	Seabream	Hepatosteatosis, alteration of lipid metabolism	(69, 71)
Triclosan (TCS)	Zebrafish	Upstream regulation of miR-125b	(68)
	Human MSCs	decreasing *aP2, lpl*, and *adipoq* gene expression	(47)
	Zebrafish	impaired mRNA expression levels of β-oxidation transcripts and lipid β-oxidation genes, including *pparα, cpt1, lpbe, cyp4a10*, and *aco*	(55)
Tyhriphenyltin (TPT)	Trout Primary adipocite culture	Promotion of adipocytes differentiation by enhancing PPARγ and C/EBPα protein expression	(60)
tris (1,3-dichloroisopropyl) phosphate (TDCIPP)	Zebrafish	Obesity induction and alteration of circadian rhythms	(57)

The integration of results suggests that hepatic steatosis, the first signal of the onset of several metabolic diseases, easily occurs following the exposure to environmental concentrations of pollutants, thus triggering an increase of FA synthesis or uptake and their decreased oxidation. Widespread pollutants including BPA, phthalates, PFCs, POPs, and TBT, targeting NR, induce FA synthesis in different animal models, highlighting the activation of a common pathway mediating the toxicity among species. The main evidence consists in the fact that liver also mediates xenobiotic metabolism which may increase oxidative stress and in turn impacts the correct FA metabolism. However, since few evidence still exists to characterize pathways and patterns leading to altered functional development, all these results should be considered and integrated by Environmental Agencies to propose novel biomarkers and innovative endpoints for the development of novel Organization for Economic Co-operation and Development (OEDC) test guidelines to screen chemical danger for metabolic functions.

## Author contributions

All authors listed have made a substantial, direct and intellectual contribution to the work, and approved it for publication.

### Conflict of interest statement

The authors declare that the research was conducted in the absence of any commercial or financial relationships that could be construed as a potential conflict of interest.
